# Evaluation of cyanotoxin L-BMAA effect on α-synuclein and TDP43 proteinopathy

**DOI:** 10.3389/fimmu.2024.1360068

**Published:** 2024-03-26

**Authors:** Paola Sini, Grazia Galleri, Cristina Ciampelli, Manuela Galioto, Bachisio Mario Padedda, Antonella Lugliè, Ciro Iaccarino, Claudia Crosio

**Affiliations:** ^1^ Laboratory of Molecular Biology, Department of Biomedical Sciences, University of Sassari, Sassari, Italy; ^2^ Laboratory of Ecology, Department of Architecture, Design and Urban Planning, University of Sassari, Sassari, Italy

**Keywords:** L-BMAA, cyanotoxins, TDP43, α-synuclein, ALS, PD

## Abstract

The complex interplay between genetic and environmental factors is considered the cause of neurodegenerative diseases including Parkinson’s disease (PD) and Amyotrophic Lateral Sclerosis (ALS). Among the environmental factors, toxins produced by cyanobacteria have received much attention due to the significant increase in cyanobacteria growth worldwide. In particular, L-BMAA toxin, produced by diverse taxa of cyanobacteria, dinoflagellates and diatoms, has been extensively correlated to neurodegeneration. The molecular mechanism of L-BMAA neurotoxicity is still cryptic and far from being understood. In this research article, we have investigated the molecular pathways altered by L-BMAA exposure in cell systems, highlighting a significant increase in specific stress pathways and an impairment in autophagic processes. Interestingly, these changes lead to the accumulation of both α-synuclein and TDP43, which are correlated with PD and ALS proteinopathy, respectively. Finally, we were able to demonstrate specific alterations of TDP43 WT or pathological mutants with respect to protein accumulation, aggregation and cytoplasmic translocation, some of the typical features of both sporadic and familial ALS.

## Introduction

Neurodegenerative diseases (NDs) comprise a wide range of pathological conditions characterized by alterations in common pathways, ranging from altered protein metabolism, mitochondrial dysfunction, oxidative stress to inflammation (1). Despite advancements, the origins and progression of most neurodegenerative diseases remain largely elusive. These diseases are broadly categorized into sporadic (sND) and familial (fND) with around 5-10% having a clear genetic basis (fND), while the majority (sND) lacks identified genetic contributions. Consequently, sNDs are believed to result from intricate interactions between genetic factors and environmental influences across one’s lifespan. Notably, the global rise in life expectancy is closely associated with a significant surge in age-related diseases (2). Among environmental factors, exposure to cyanotoxins has emerged as a growing health threat due to their persistence and bioaccessibility in the environment (3–5). Cyanobacterial blooms, driven by anthropogenic factors, such as eutrophication, aquaculture, introduction of alien species, hydrodynamic changes in coastal systems and global climate change, have increased, impacting ecosystems and human health (6, 7). Cyanotoxins, implicated in various human diseases, are linked to seafood poisoning syndromes and respiratory or dermatological irritation upon human exposure (8, 9). Additionally, mounting epidemiological evidence suggests an association between exposure to environmental toxins and neurodegenerative diseases, including Amyotrophic Lateral Sclerosis (ALS), Alzheimer disease (AD) and Parkinson Disease (PD) (10–13), particularly ALS/Parkinsonism Dementia Complex (ALS/PDC) found on the islands of Guam (reviewed (5, 14)). Initial links between ALS/PDC and the neurotoxin L-BMAA, produced by cyanobacteria, were proposed by Spencer and colleagues, pointing to its presence in cycad seeds used in Guam’s food and traditional medicine (15) (16). L-BMAA is produced by almost all known groups of cyanobacteria, including cyanobacterial symbionts (e.g. Nostoc) and free-living cyanobacteria (e.g. Anabaena, Microcystis), marine diatoms (e.g. Navicula, Skeletonema) and dinoflagellates (e.g. Gymnodinium) in a wide variety of ecosystems worldwide (17–19).

Subsequent experimental findings validated L-BMAA neurotoxicity in both human and animal model. L-BMAA was found, in postmortem brain tissues from ALS and PD’s patients (20–22); induced motor-system diseases in monkeys (23–25),; cell death and astrogliosis in mice, accompanied by TDP-43 cytoplasmic accumulation (26). Chronic low-dose BMAA exposure, combined with low expression of ALS TDP-43 mutation (Q331K) expression, resulted in motor phenotype potentially involving the unfolded protein response (UPR) pathway (27). Finally, L-BMAA toxicity was confirmed in various cellular models (3, 28), though the molecular mechanisms varied among cell types (29).

L-BMAA is a non-lipophilic, non-essential amino acid present in both free and protein-bound forms. While the exact mechanism of L-BMAA toxicity remains complex, three main hypotheses have been proposed. Firstly, L-BMAA may bind to ionotropic (iGluR) and metabotropic (mGluR) receptors, suggesting excitotoxicity through excessive glutamate receptor stimulation (30).. Secondly, L-BMAA inhibits cystine/glutamate antiporter (system Xc-)-mediated cystine uptake, depleting glutathione and increasing oxidative stress (31). Thirdly, in the cytoplasm, L-BMAA may be incorporated into newly synthesized cellular proteins instead of alanine and/or serine, potentially promoting protein misfolding and the formation of insoluble aggregates typical of neurodegenerative diseases (32). Notably *in vivo* and *in vitro* experiments often use purified L-BMAA, while the mode of exposure (live cells, extracts, or purified toxins) is one of the most important factors influencing the toxicity of cyanobacteria (11, 33). For instance, several studies have reported that the effects of L-BMAA are generally more pronounced when exposure is performed with intact cells, even at low cell density, than when exposed to extracts or purified toxins (34). Furthermore, L-BMAA exposure, even at low concentrations, exacerbates the effects of other neurotoxins (35, 36). In fact, the complex mixture present in cyanobacterial biomasses may cause various additive and multiplicative effects. It is therefore difficult to apply a specific assay that would respond equally to all toxic compounds and provide relevant information for the assessment of potential human or animal toxicity.

Cyanotoxin exposure has primarily associated with ALS/Parkinsonism Dementia Complex (ALS/PDC), ALS and PD. ALS is a progressive and fatal disorder characterized by degeneration of upper and lower motor neurons. Approximately 5-10% of cases are familial (fALS), with pathogenic variants in TDP-43, C9ORF72, SOD1, and FUS being common (37–39). TDP-43, the major component of the insoluble and ubiquitinated inclusions in ALS and frontotemporal lobar degeneration (FTLD or FTLD-TDP) (40, 41), undergoes mis-localization in the cytoplasm in pathological conditions (42).

PD is the second most common neurodegenerative disease and genetic studies have identified approximately 20 different causative genes including α-Synuclein, LRRK2, PINK1, Parkin. α-Synuclein was the first mutated gene to be linked to the disease in two different PD families (43). Widespread aggregation of α-synuclein protein was then found to be the major component of Lewy bodies, the neuropathological hallmark of PD (44). To date, the function of α-synuclein is largely unknown, despite the development of many cellular and animal models (45), however, several experimental results underline a significant contribution of synuclein to vesicle trafficking (46, 47).

In this study, we explore the molecular mechanisms through which cyanobacterial extract or L-BMAA exposure may induce cellular toxicity. Interestingly, we were able to demonstrate a significant contribution of L-BMAA incorporation into newly synthesized cellular proteins to cellular toxicity and its impact on autophagy. Importantly, this impairment is associated with an accumulation of both α-synuclein and TDP43, the major misfolded proteins in PD and ALS, respectively. Finally, we extensively studied the cyanotoxin effect in the presence of TDP43 WT or pathological mutants in different models, revealing significant alterations in various TDP43 pathological signs including misfolding, aggregation and mislocalization.

## Materials and methods

### Antibodies and reagents

The following primary antibodies were used in this study: Myc monoclonal antibody (M4439, Sigma-Aldrich, Merk KGaA, Darmstadt, Germany, 1:1000), β-actin antibody (A5441, Sigma-Aldrich, Merk KGaA, 1:5000), TARDBP (190782-2-AP, Proteintech Europe Manchester, UK, 1:1000), histone H4 (SAB4500313, Merk KGaA, 1:2000), caspase-3 (9665, Cell Signaling Technology, Danvers, Massachusetts, USA 1:1000), LC-3B (2775, Cell Signaling Technology, 1:1000), p62 (GTX100685, GeneTex Inc, Irvine, California USA, 1:500), anti-rabbit peroxidase-conjugated secondary antibody (AP132P, Merk KGaA, 1:3000) and anti-mouse peroxidase-conjugated secondary antibody (AP124P, Merk KGaA, 1:3000); anti-rabbit, anti-mouse Alexa 488 (A-11001, Thermo Fisher Scientific, Waltham, Massachusetts, USA, 1:1000) or 647-conjugated secondary antibody (A-21244, Thermo Fisher Scientific, 1:1000). All antibodies were used at the dilution recommended by the manufacturer’s instructions.

L-BMAA, (+)-L-β-N-Methyl-αβ-diaminopropionic acid hydrochloride (B-107, Sigma-Aldrich, Merk KGaA), L-Alanine (05129, Sigma-Aldrich, Merk KGaA), L-Serine (S4500, Sigma-Aldrich, Merk KGaA), Puromycin (P8833, Sigma-Aldrich, Merk KGaA).

### Cell lines

SH-SY5Y neuroblastoma cells (CRL-2266, ATCC, Rockville, MD) and SH-SY5Y-pCHOP cells were cultured in DMEN/F12 (Thermo Fisher Scientific) supplemented with 10% fetal calf serum (FCS, Thermo Fisher Scientific). The SH-SY5Y-pCHOP cell line was generated by stable transfection of the plasmid expressing zsGreen under the control of the ER stress-responsive promoter of DNA damage-inducible transcript 3, also known as the C/EBP homologous protein (CHOP) gene. The plasmid construct was generated by cloning the CHOP (Gene ID: 1649) promoter region from -954 to +91 into the SacI and HindIII sites of the pZsGreen1-1 plasmid (Takara Bio Inc, Kusatsu, Japan). Individual clones were selected using 400 µg/ml of G1418 (Gibco, Thermo Fisher Scientific) and analyzed for their response to ER stress.

Human Embryonic Kidney (HEK) 293T cell line (CRL 3216 HEK 293T ATCC, Rockville, MD) and primary cutaneous fibroblasts from healthy individuals, from patients with sporadic ALS (carrying a heterozygous 1144G-A transition in exon 6 of the TARDBP gene, resulting in an A382T substitution in TDP-43-encoding gene) and patients with familiar ALS without mutations in the most common ALS- related genes (48) were cultured in DMEM (Thermo Fisher Scientific) supplemented with 10% foetal calf serum. All cells were grown in an incubator at 37°C, with a humidified atmosphere containing 5% CO2. Trypsin (0.5 µg/ml, 68 mM EDTA, Thermo Fisher Scientific) was added to splitting cells and then diluted in fresh medium.

### Adenovirus transduction adenovirus for TDP-43

Expression and the transduction protocol were previously described (49). SH-SY5Y were plated at 40% of confluence and, the day after, were transduced by recombinant adenovirus for 1 h in a serum-free medium. After 1 h, the medium was replaced by a medium containing 1% serum. At the indicated time points, the cells were washed twice by cold PBS 1X and lysed in Laemmli buffer 1X.

### Lake Coghinas strain *Microcystis aeruginosa* crude extracts

*Microcystis auroginosa* isolated from artificial lake Coghinas (Sassari, Italy), were cultured at adequate temperature and light condition (21 ± 1°C with a photoperiod of 16:8 light:dark cycle and irradiance of 50–70 mol photons m-2 s-1). Cell abundance was determined in subsamples (5–10 mL) of fixed samples using an inverted microscope (Axiovert 25, Zeiss, Oberkochen, Germany). 1x 10^6^/ml cells were centrifugated and washed with cold PBS 1X than the obtained pellet was homogenized and sonicated in 1 ml of cold water according to (50–52) After 3 cycles of freeze and thaw the supernatant was collected and the protein quantification of crude extract was measured with Thermo Scientific NanoDrop 2000 Spectrophotometer. The optical density of the sample at 730 nm was used to determine cell destruction.

### L-BMAA hydrochloride and LCS-MaCe treatments

Cells were plated at 60% confluence. After 16 h growth medium was removed, cells were washed with PBS1x and the indicated treatment was added in MEM (Thermo Fisher Scientific) supplemented with 1% FBS and incubated for different exposure times.

### Assessment of cell viability

Cell viability was determined by an MTS assay (CellTiter 96 Aqueous One Solution Reagent, Promega, Madison, Wisconsin, USA) according to the manufacturer’s instructions. Absorbance at 490 nm was measured in a multilabel counter (Victor X5, PerkinElmer, Waltham, Massachusetts, USA).

### Cellular ROS assay

Measurement of produced intracellular reactive oxygen species was determinate with a commercial kit (DCFDA - Cellular ROS Assay Kit/Reactive Oxygen Species Assay Kit, ab113851, Abcam, Cambridge, UK) according to the manufacturer’s protocol. DCFDA is deacetylated by cellular esterases to a non-fluorescent compound, which is later oxidized by ROS into 2’,7’–dichlorofluorescein (DCF). DCF was detected by fluorescence spectroscopy with excitation/emission at 485 nm/535 nm in a multilabel counter (Victor X5, PerkinElmer).

### GFP-autofluorescence measurement

Stable cell line SH-SY5Y-pCHOP, expressing zsGreen under the control of CHOP promoter were plated 1*10^^5^ in a 24-multiwell plate and transduced or not with adenoviral particles coding for TDP43^WT/M337V/A382T^ vectors. 24 hours after infection cells were treated with LCS-MaCe total extract or L- BMAA in base growth media Dulbecco MEM/F12 medium containing 10% FBS without phenol red. Following 24h of treatments, GFP fluorescence was detected with excitation/emission at 485 nm/535 nm of 1s in a multi-reader plate (Victor X5, PerkinElmer).

### SDS PAGE and Western immunoblotting

Protein content was determined using Bradford protein assay (27813, Sigma-Aldrich, Merk KGaA). Uniform amounts of protein extracts were loaded by standard SDS/PAGE. Samples were then electroblotted on Pierce Nitrocellulose Membrane, 0.45 µm (Thermo Fisher Scientific). Then, membranes were incubated in a blocking solution of 3% low-fat milk, diluted in PBS 1X-Tween 0.05% solution with the indicated primary antibody for 16 h at 4°C. Secondary antibody were used to reveal immunocomplexes using LiteUP WB Chemiluminescent Substrate (EMP002005, Euroclone, Hercules, California, USA). Chemiluminescent imaging was obtained using ChemiDoc XRS+ System (Bio-Rad Laboratories, Hercules, California, USA). The apparent molecular weight of proteins was determined by calibrating the blots with pre-stained molecular weight markers (Bio-Rad Laboratories).

### Fractionation and biochemical analysis

Treated cells were washed twice with cold PBS 1X, lysed in cold RIPA buffer (50 mM Tris- HCl, pH 8, 150 mM NaCl, 1% NP-40, 0.1% SDS, 0.5 mM sodiumdeoxycholate) supplemented with 1x Protease inhibitor cocktail (Complete™, Mini, EDTA-free Protease Inhibitor Cocktail, Sigma-Aldrich, Merk KGaA), a mixture of phosphatase inhibitors, 1 mM NaF, 1 mM Na_3_VO_4_, (Sigma-Aldrich, Merk KGaA) and sonicated. Lysates were centrifuged (twice to prevent contamination caused by carrying over) for 30’ at 100,000*g at 4°C, the pellets were re-sonicated and re-centrifuged at 100,000*g for 30’ at 4°C and the supernatant was collected as RIPA buffer soluble fraction. RIPA buffer-insoluble pellets were dissolved in urea buffer (7 M urea, 2 M thiourea, 4% CHAPS, 30 mM Tris- HCl, pH 8.5) and sonicated. Soluble and insoluble fractions were subsequently analyzed by immunoblotting.

### Immunofluorescence

Cells were plated at 2*10^5 in 24-multiwell plates on 12mm glass coverslips, fixed with 4% paraformaldehyde in PBS 1X and permeabilized with 0.2% Triton X-100 in PBS 1X for 15’. After a blocking step of 1 h in 5% BSA, diluted in PBS 1X–0.05% Tween-20, cells were incubated with the indicated primary antibody diluted in blocking solution, overnight at 4°C, and then incubated with the proper secondary antibody, diluted in blocking solution, for 1h at RT; DAPI (D9542, Sigma Aldrich, Merk KGaA), fluorescent staining of DNA content and nuclei, was added diluted 1∶1000 in blocking solution. Cells were then analyzed with a Leica TCS SP5 confocal microscopy, with LAS (Leica Application Suite) lite 170 image software (Advance Fluorescence 2.7.3.9723). Using the 3D viewer in LAS Lite 170, we visualize and analyze three-dimensional datasets acquired through confocal microscopy.

### FACS analysis

Cells treated as indicated were diluted in cold 1XPBS, centrifuged and resuspended in 1XBinding buffer (10 mM HEPES, 140 mM NaCl, 2.5 mM CaCl2, pH 7.4) at the concentration of 10^6cells/mL. Subsequently, Annexin V-FITC staining solution and Propidium iodine (PI) staining solution were added at the final concentration 20 μg/mL. Cells were then analyzed using a flow cytometer FACSCanto (Becton Dickinson, Franklin Lakes, NJ, USA). Flow cytometer acquired 3x10^4^ total events. Data analysis was carried out using BD FACSDivaTM software 6.1.3.

### Statistical analysis

The results are presented as means ± SD of independent experiments as indicated. For bands analysis in Western blot experiments, after image acquisition, the protein bands were quantified by densitometry and normalized to the specific loading control using Quantity One software (Version 4.6.8, Bio-Rad Laboratories). Statistical evaluation was conducted by one-way ANOVA and Bonferroni’s multiple comparison post-test. Values significantly different from the relative control are indicated with *, **, or *** symbols when p < 0.05, p < 0.01, and p < 0.001, respectively.

## Results

### Effects of L-BMAA or cyanobacteria crude extracts on neuronal cell line SHSY5Y

The causal relationship between neuronal degeneration and exposure to cyanotoxins, such as L-BMAA, is still quite controversial. To establish a reliable neurotoxicity assessment model, we opted to employ the human-derived neuroblastoma cell line SH-SY5Y. These cells, derived from a human metastatic neuroblastoma, have dopaminergic, cholinergic, glutamatergic and adenosinergic properties and are widely used as a model to study NDs (53). As mentioned in the introduction toxicity of cyanobacteria can be influenced by the mode of exposure; To mimic natural exposure to neurotoxins, we utilized both pure synthetic L-BMAA and crude cellular extracts obtained from a strain of Microcystis aeruginosa isolated from Coghinas Lake (LCS-MaCe, in which L-BMAA was measured, maximum 17.84 μg L-1 (54),), mimicking natural exposure to neurotoxins. LCS-MaCe was obtained using the freeze-thaw method, that is considered a standard method to lyse cells and it is recommended by U.S. Environmental Protection Agency (EPA) Method 546 (50, 51).

**Figure 1:**
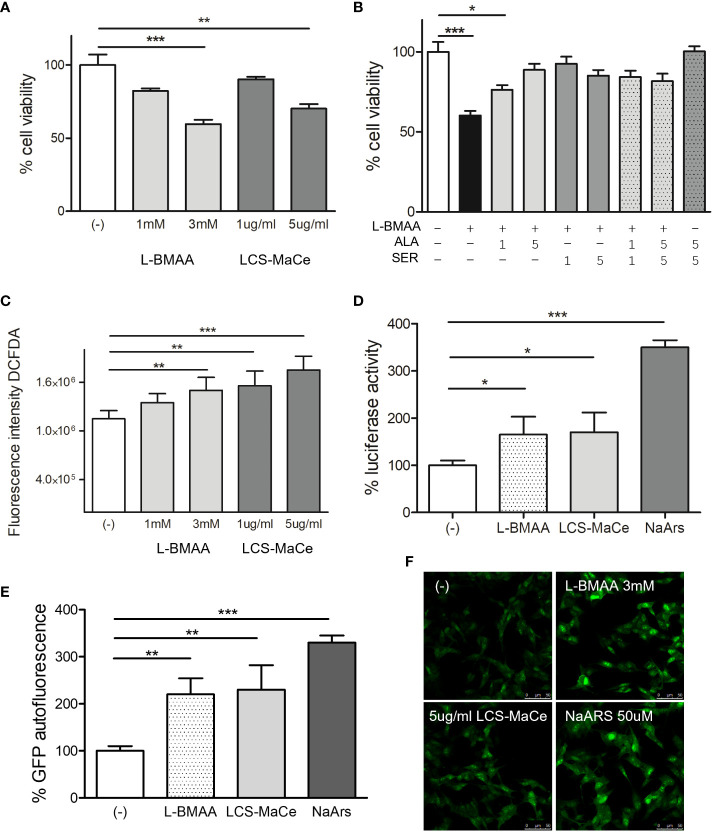
Effects of L-BMAA and LCS-MaCe exposure on SHSY-5Y neuronal cells. **(A)** Dose-dependent reduction in cell viability of SHSY-5Y cells exposed to different concentrations of pure L-BMAA (1 or 3 mM) or LCS-MaCe (1 or 5 µg) for 24 h, measured by MTS assay. **(B)** MTS assay on cells as in **(A)** exposed to 3mM l-BMAA, in combination with different doses (1 or 5mM) of the amino acids serine (SER), alanine (ALA) or both **(C)** SHSY-5Y cells were treated as in **(A)** and cellular ROS were measured using DCFDA. The signal was detected with excitation/emission at 485 nm/535 nm of 1s in a multi-reader plate (Victor X5, PerkinElmer). **(D)** SHSY-5Y were transfected with pARE-Luc and Renilla and 24h later treated as in **(A)**. Luciferase activity was measured in a multiplate reader using the Dual-GlowTM Luciferase Assay System (Promega, USA). Firefly luciferase activity was then normalized to Renilla luciferase activity to control for transfection efficiency. Data were then normalized to luciferase activity in cells transfected with empty vector, which was assigned a value of 1 **(E)** SH-SY5Y-pCHOP cells were treated as in **(A)** and GFP fluorescence was detected with excitation/emission at 485 nm/535 nm of 1s in a multi-plate reader (Victor X5, PerkinElmer). **(F)** Analysis of GFP autofluorescence in the indicated cells was performed using a Leica TCS SP5 confocal microscope. NaArs 10 µM treatment for 24 h was used as a controlData in **(A-E, L)** are the mean and standard deviation ( ± SD) of at least three independent experiments. *, P < 0.05; **, P < 0.01, ***, P < 0.005.

In preliminary experiments testing different protocols, doses, and exposure times for L-BMAA (data not shown) we observed a reduction in cell viability when SH-SY5Y cells were exposed to increasing concentrations of L-BMAA or LCS-MaCe and tested by MTS assays (**Figure 1A**). Both treatments induce a significant reduction in cell viability, which can be rescued by adding to cell medium the aminoacids L-serine or alanine (**Figure 1B**).

To discern the cause of the reduction in the observed cell viability we evaluated oxidative or ER stress.

Exposure to the pure toxin L-BMAA or to LCS-MaCe caused a significant increase in oxidative stress in SH-SY5Y cells, as assessed by measuring both cellular ROS levels (**Figure 1C**) and transcriptional activation of the antioxidant responsive element (ARE), a key regulatory element of many cellular defense enzymes, that drives the luciferase reporter expression (**Figure 1D**).

ER stress was assessed evaluating gene activation of C/EBP homologous protein (CHOP), one of the components of the ER-stress-mediated apoptosis pathway. Under physiological conditions, CHOP is expressed at low levels and localizes in the cytoplasm; however, under stress conditions, an up-regulation of CHOP mRNA levels and its protein accumulation are observed (55, 56). To monitor CHOP activation, we took advantage of a stable cell line SH-SY5Y-pCHOP expressing zsGreen (Zoanthus sp. human codon-optimized green fluorescent protein 1) under the control of the human CHOP promoter, previously generated in our laboratory.

GFP autofluorescence corresponding to CHOP transcriptional activation, was measured after L-BMAA or LCS-MaCe exposure by both fluorescence measurement by multi plate reader (**Figure 1E**) and autofluorescence analysis by confocal microscope (**Figure 1F**). Notably, the observed increase in GFP autofluorescence is comparable to the intensity induced by Sodium Arsenite, a known inducer of acute stress (57).

### Cellular responses to cyanotoxin exposure: autophagy and proteinopathy

**Figure 2:**
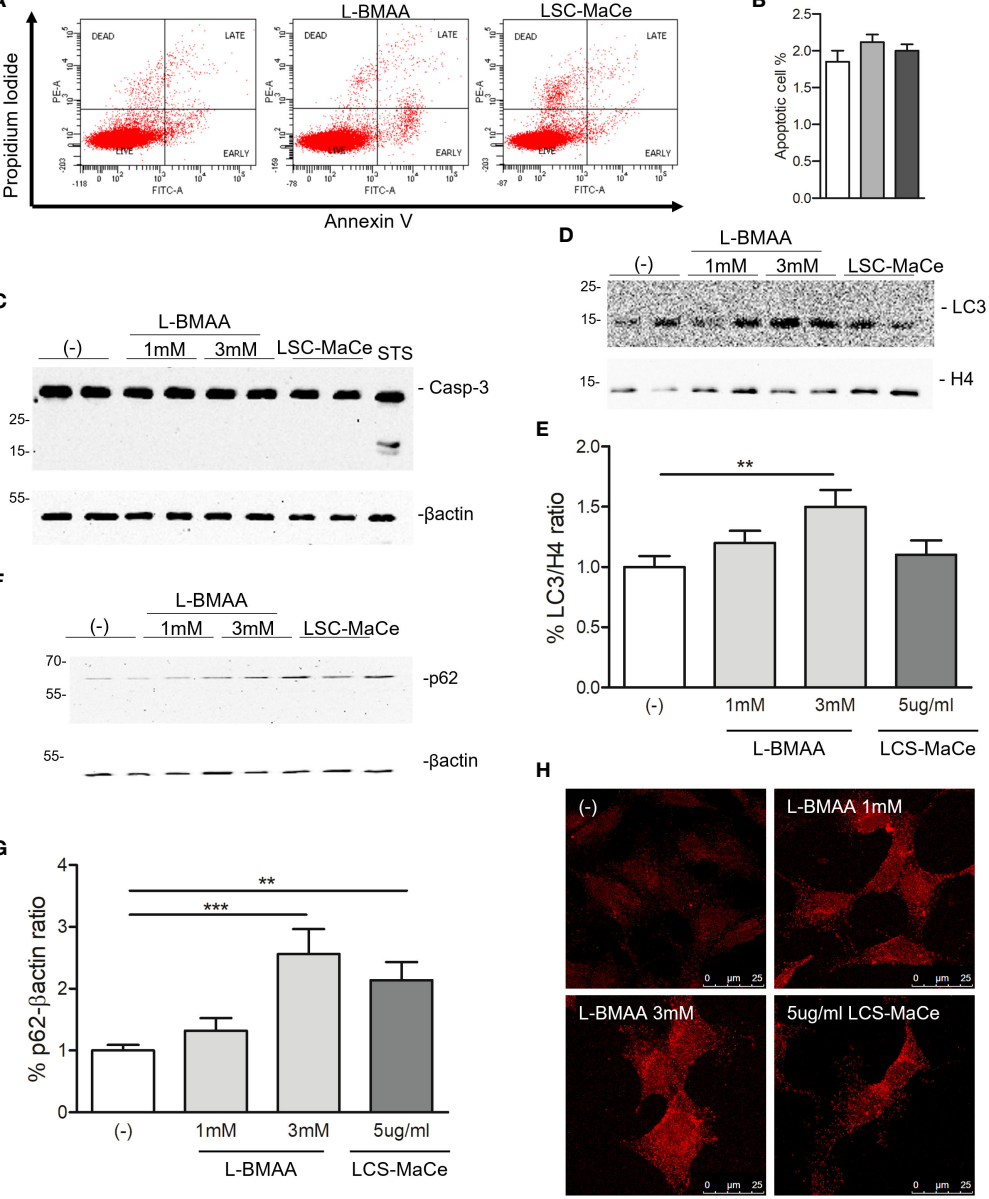
Evaluation of apoptosis and autophagy in SHSY-5Y neuronal cells exposed to L-BMAA and LCS-MaCe. **(A)** SHSY-5Y cells were exposed to pure L-BMAA 3 mM or LCS-MaCe 5 µg/ml for 24 h. Cells were labelled with Propidium Iodide (PI) and Annexin V to assess the apoptotic and necrotic phases. Cells were analyzed by flow cytometry on FACSCanto™ using FACSDiva software. **(B)** Quantification of results in A) **(C)** Western blot analysis on total protein extracts, obtained from SHSY-5Y cells treated with L-BMAA 1 or 3 mM, or with LCS-MaCe 5µg/ml for 24h, using the apoptotic markers anti-caspase3. Treatment with staurosporine (STS) for 6h was used as positive control to induce caspase-3 cleavage. Anti-βactin was used as an equal loading control. Western blot analysis of cells as in **(C)** using the autophagy markers anti-LC3B **(D)** and anti-p62 **(F)**. Anti-H4 and anti-βactin were used as equal loading control. **(E)** Quantification of results in D) using ChemiDOC XRS+ system with Quantity One™ software. **(G)** Quantification of results in F) using ChemiDOC XRS+ system with Quantity One™ software. **(H)** Cells as in **(C)** were analyzed by immunofluorescence. Endogenous p62 signal was detected by primary anti-p62 antibody and secondary goat anti-rabbit IgG Alexa Fluor^®^ 647; cells were analyzed using a Leica TCS SP5 confocal microscope. Data in **(B-E, G)** are the mean and standard deviation ( ± SD) of at least three independent experiments. *, P < 0.05; **, P < 0.01, ***, P < 0.005. In order to minimize the variability of the experimental results, each data point in panels **(C, D, F)** consists of two biological replicates.

Since both oxidative and ER stress can induce cell death, a flow cytometry analysis for necrosis, apoptosis was performed labeling the cells by propidium iodide (PI) and Annexin V. The results indicated that neither apoptosis nor necrosis appeared to be the primary determinant of the effects of L-BMAA or LCS-MaCe (**Figures 2A, B**). These data were confirmed by Western blot (**Figure 2C**) where no Caspase-3 cleavage was detected by the different treatment compared to staurosporin (STS) treatment used as positive control. On the contrary, when we evaluated two autophagic markers, LC3B and p62, we were able to highlight a specific induction by L-BMAA or LCS-MaCe treatments (**Figures 2D–H**), indicating an impairment of the autophagic flux (58).

**Figure 3:**
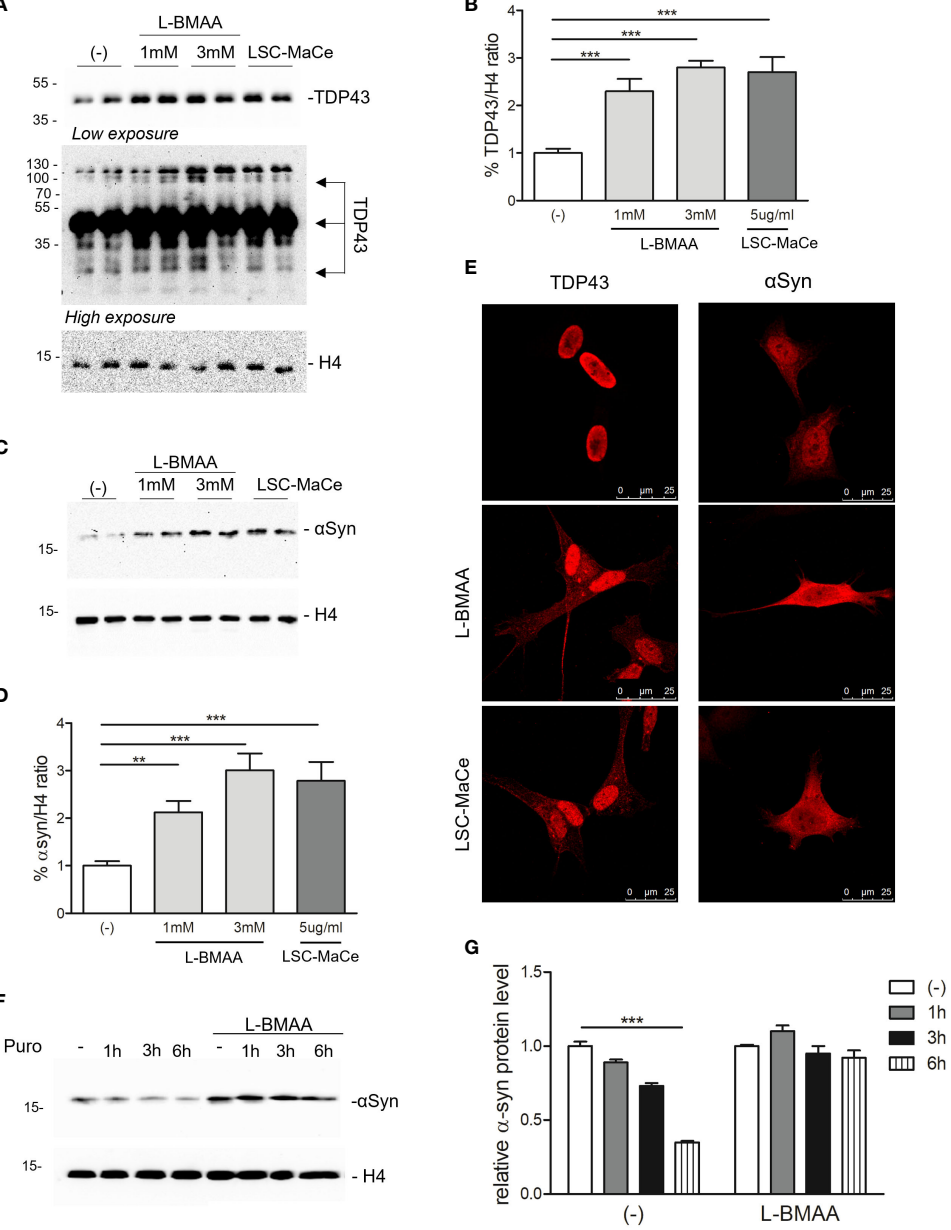
Effects of L-BMAA and LCS-MaCe exposure on the ALS-causing gene TDP-43 and on the PD-causing gene α-synuclein. **(A)** SHSY-5Y cells were exposed to L-BMAA (1 or 3mM) or LCS-MaCe 5 µg/ml for 24h. Total cell lysates were subjected to reducing SDS-PAGE and Western blot. Anti-TDP-43 antibody was used to visualize TDP-43 expression, anti-βactin as an equal loading control. **(B)** Quantification of results in **(A)** using ChemiDOC XRS+ system with Quantity One™ software. **(C)** SHSY-5Y cells were plated 1*10^5 and transduced with adenoviral particles encoding α-synuclein. 24h after transduction, cells were treated as in **(A)** and total cell lysates were analyzed by immunoblotting using anti-α-synuclein antibody and anti-β-actin as loading control **(D)** Quantification of results in **(C)** using ChemiDOC XRS+ system with Quantity One™ software. **(E, F)** Immunofluorescence analysis on SHSY-5Y treated as in **(A)** using anti-TDP-43 antibody and on SH-SY5Y were transduced and treated as in **(C)** using anti- α-synuclein antibody. Both primary antibodies were revealed using anti-rabbit ALEXA 546 secondary antibody. The slides were analyzed by Leica confocal microscope. **(F)** SH-SY5Y were transduced and treated as in **(C)**. After 24h puromycin 10µg/ml was added and cells collected at different time points (1h, 3h, 6h). Cell lysates were subjected to reducing SDS-PAGE and western blot. The anti-αSynuclein antibody was used to visualize α-synuclein expression. βactin serves as controls for equal loading of samples. **(G)** Quantification of results in **(E)** using ChemiDOC XRS+ system with Quantity One™ software. Data in **(B-E, G)** are the mean and standard deviation ( ± SD) of at least three independent experiments. *, P < 0.05; **, P < 0.01, ***, P < 0.005. In figure **(C, D, F)** each experimental data point is duplicated to minimize variability.

Since autophagy is a catabolic process for unnecessary or dysfunctional cytoplasmic contents mediated by lysosomal degradation pathways and autophagy mediates both TDP43 (59) and α-synuclein turnover (60), two of the major components of protein aggregates in degenerating neurons respectively in ALS and PD, we evaluated the effects of L-BMAA or LCS-MaCe on the protein levels of TDP-43 and α-synuclein. Exposure to cyanotoxins increases the endogenous TDP-43 protein level, and at high exposure times we were able to also demonstrate an accumulation of high molecular weight TDP-43 (**Figures 3A, B**). Furthermore, confocal analysis in SH-SY5Y cells revealed a moderate cytoplasmic TDP-43 relocalization (**Figure 2E**, left column), another typical sign of TDP43 proteinopathy.

To evaluate the effects of L-BMAA or LCS-MaCe on α-synuclein, since this protein is barely detectable in SH-SY5Y cells, we overexpressed it by adenoviral transduction (61). Western blot analysis shows a significant accumulation of α-synuclein (**Figures 2C, D**) upon L-BMAA or LCS-MaCe exposure. To assess whether the observed α-synuclein accumulation was due to a defect in protein turnover, we performed a treatment with puromycin, a drug that inhibits translational elongation, in presence or absence of L-BMAA. Cells were treated or not with L-BMAA for 24h and then with puromycin for the indicated time. Notably, cells treated with L-BMAA and puromycin exhibited a significant reduction in α-synuclein turnover compared to the cells untreated by L-BMAA (**Figures 2F, G**). At 6 hours in L-BMAA untreated cells more than 50% of α-synuclein has been degraded while no differences are detectable in L-BMAA treated cells (**Figure 2G**). Conversely, the expression of β-actin remained unaffected by the treatments, serving as a control for protein expression in the cells. Finally, accumulation of α-synuclein accumulation was confirmed by confocal analysis (**Figure 2E** -right column).

In summary, our findings indicate that exposure to L-BMAA or LCS-MaCe impacts autophagic pathways and replicates various molecular features typical of neurodegeneration, including the accumulation of TDP43 and α-synuclein.

### Effect of L-BMAA or cyanobacteria crude extracts on cellular models for ALS

Considering that ALS is generally thought to progress as a consequence of genetic susceptibility and environmental influences, and that TDP-43 has been observed as the major component of ubiquitinated inclusions in post-mortem tissues of ALS patients and patients with frontotemporal dementia (41), we decided to extend our analysis by evaluating the effects of L-BMAA or LSC-MaCe exposure in different ALS cellular models (49).

**Figure 4:**
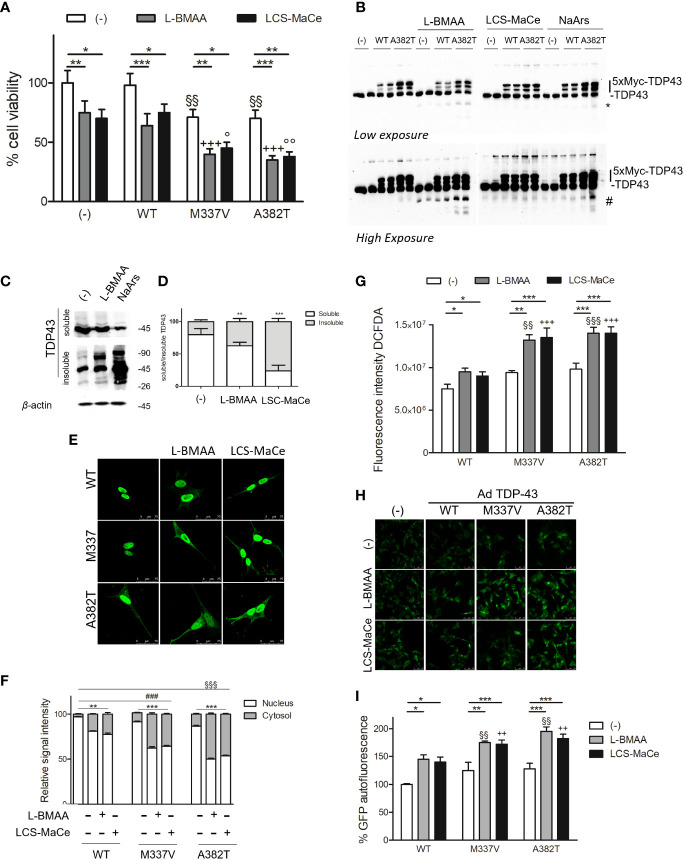
L-BMAA and LCS/MaCe treatments exacerbate the pathological TDP-43 phenotype. **(A)** SHSY-5Y cells were plated 1*10^5 and transduced with adenoviral particles encoding TDP43 WT or carrying the pathological mutation M337V or A382T. 24h after transduction, cells were exposed to L-BMAA 3mM or LCS-MaCe 5 µg/µl for another 24h. Cell viability was assessed by MTS assay. **(B)** Total cell lysates were obtained from cells as in **(A)** and subjected to reducing SDS-PAGE and Western blot. Anti-TDP-43 antibody was used to visualize TDP-43 expression, anti-βactin as an equal loading control. **(C)** Biochemical fractionation of SHSY5Y cells treated as in **(A)** and cell lysates were separated into soluble and insoluble fractions and subsequently analyzed by immunoblotting. Anti-TDP-43 antibody was used to visualize TDP-43 expression and aggregation, β-actin as a control for equal loading of samples and correct fraction separation. **(D)** Quantification of results in **(B)** using ChemiDOC XRS+ system with Quantity One™ software. **(E)** Cells as in **(A)** were analyzed by immunofluorescence using an anti-myc. **(F)** Quantification of the data in **(D)**. **(G)** Oxidative stress measured by measured using DCFDA. **(H)** ER stress analyzed by CHOP promoter induction in SH-SY5Y-CHOP-GPF stable clones as in **Figure 1F** **(I)** Quantification of GFP autofluorescence as represented in **(H)**. Data in A-B-C-D-E and L are the mean and standard deviation ( ± SD) from at least three independent experiments. *, P < 0.05; **, P < 0.01, ***, P < 0.005. * respect to untreated of the same genotype, § respect to L-BMAA treatment among the different genotypes, + respect to LCS-MaCe treatment among the different genotypes. In order to minimize the variability of the experimental results, each data point in panel **(B)** consists of two biological replicates.

SH-SY5Y cells were transduced with adenoviral particles encoding WT or pathological TDP-43 mutant (A382T or M337V) and then exposed to varying doses of L-BMAA neurotoxin or LSC-MaCe. Confirming previous findings (49), a reduction in cell viability was observed following adenoviral delivery of myc-tagged mutant TDP-43 in SH-SY5Y neuronal cells (**Figure 4A**). Simultaneous exposure to increasing doses of L-BMAA or LSC-MaCe exacerbated the toxicity induced by both pathological variant A382T and M337V (**Figure 3A**). Analysis of transduced and basal TDP-43 expression through Western blot revealed characteristic bands, with higher molecular weights indicative of the 5X-Myc tag and lower bands suggesting degradation (**Figure 4B**). Interestingly, L-BMAA treatment determines the formation of TDP 43 degradation products for both transduced (asterisks in **Figure 4B**) and endogenous TDP43 (**Figure 4B** high exposure). Further characterization of the L-BMAA effect on TDP43 proteinopathy involved biochemical fractionation, highlighting a significant increase in insoluble TDP-43, a well-known marker of ALS (**Figures 4C, D**). Since TDP-43 is known to shuttle between the nucleus and cytoplasm and to form cytoplasmic protein aggregates under pathological conditions, we performed immunostaining using anti-myc tag antibody that only labels exogenous TDP-43. As shown in **Figure 4E** and quantified in **Figure 4F**, exposure to both pure L-BMAA or LCS-MaCe induces a stronger delocalization of mutant TDP-43 to the cytoplasm. Finally, we observe an exacerbation of both oxidative (**Figure 4G**) and ER stress (**Figures 4H, I**) in the presence of both mutant TDP43 and cyanotoxins.

Collectively our experimental data strongly suggest that in cellular ALS experimental models, exposure to L-BMAA or crude extracts from cyanobacteria can induce cellular stress comparable to the expression of pathological TDP-43 variants, ultimately exacerbating their phenotype in cellular ALS models.

### Analysis of L-BMAA or LCS-MaCe exposure in primary human fibroblast from both sALS and fALS

**Figure 5:**
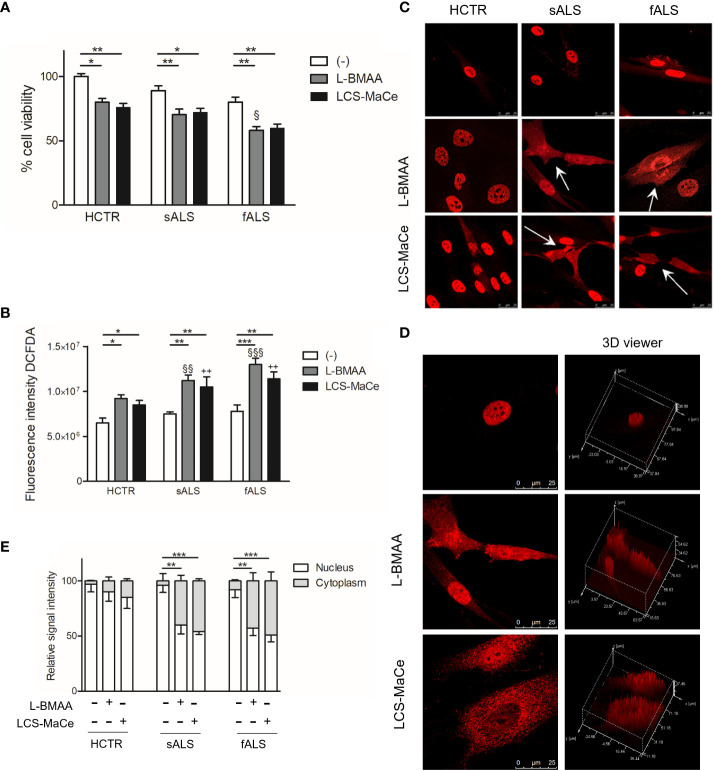
L-BMAA and LCS/MaCe treatments exacerbate the pathological phenotype in primary cutaneous fibroblasts from ALS patients. Primary cutaneous fibroblasts from healthy donors (CTR) or patients with sporadic ALS (sALS) or familial ALS (fSLA, TDP-43A382T) were exposed to L-BMAA 3mM or LCS-MaCe 5µg/µl for 24h. **(A)** Cell viability was assessed by MTS assay. **(B)** Cellular ROS were measured using DCFDA. The signal was detected with excitation/emission at 485 nm/535 nm of 1s in a multi-reader plate (Victor X5, PerkinElmer). **(C)** Protein localization was analyzed by immunofluorescence. TDP-43 signal was detected by primary anti-TDP-43 antibody and secondary goat anti-rabbit IgG Alexa Fluor^®^ 647. Cells were analyzed using a Leica TCS SP5 confocal microscope. **(D)** Analysis of intracellular TDP-43 distribution with LAS lite 170 image software (Leica) using 3D viewer tool **(E)** Graphical representation of the data in **(C)** * respect to untreated of the same genotype, § respect to L-BMAA treatment among the different genotypes, + respect to LCS-MaCe treatment among the different genotypes. **, P < 0.01, ***, P < 0.005.

To delve deeper into the influence of cyanotoxins on cellular functions, we used fibroblasts from both ALS patients and healthy controls. These fibroblasts from ALS patients provide a valuable platform for studying environmental triggers in a genetic background more closely resembling sporadic ALS (sALS). Additionally, fibroblasts exhibit characteristics relevant to TDP-43 protein metabolism and abnormal accumulation (62, 63). In light of these considerations, primary cutaneous fibroblasts from healthy donors, from patients with sporadic ALS (carrying a heterozygous 1144G-A transition in exon 6 of the TARDBP gene, resulting in an A382T substitution in the TDP-43 encoding gene) or from patients with familial ALS without mutations in the most common ALS-related genes were studied to elucidate the mechanisms of L-BMAA toxicity (64). Initial assessments of cell viability, both at low and high concentrations (1mM or 3mM), of L-BMAA or LCS-MaCe treatments after 24 hours, did not yield statistically significant decreases, although a noticeable trend was observed (data not shown). Subsequently, a time-course experiment revealed a statistically significant reduction in viability, particularly in fibroblasts from familial ALS patients carrying the mentioned TARDBP gene transition after 72h of treatment. (**Figure 5A**).

Furthermore, an escalation in oxidative stress was measured upon L-BMAA or LCS-MaCe exposure (**Figure 5B**), in all fALS and sALS fibroblasts, respect to healthy controls.

Subsequent evaluation of TDP-43 nuclear/cytoplasmic localization through immunofluorescence and confocal analysis unveiled a significant change in TDP-43 localization (**Figure 5C**). Abnormal cytoplasmic relocalization of TDP-43, predominantly affecting fibroblasts from ALS patients, was observed (highlighted with white arrows in **Figure 5C**, quantification in **Figure 5E**). Treatment with both L-BMAA and LCS-MaCe induced features similar to those observed in TDP-43-overexpressing models: nuclear depletion and cytoplasmic delocalization. While cyanotoxin exposure impacted healthy control fibroblasts, TDP-43 delocalization and abnormal accumulation in the cytoplasm were significantly amplified in cells from ALS patients.

## Discussion

The documented presence of cyanobacteria, including cyanotoxin-producing species, in water bodies and their blooms pose a potential risk to human health. Recent epidemiological correlations linking various neurological diseases, including ALS and PD, to toxic cyanobacteria -especially those producing neurotoxins such as L-BMAA-, prompted us to an extensive exploration into the intricate molecular mechanisms by which cyanobacterial extracts, or pure toxin L-BMAA, may inflict neuronal damage in cellular models. As highlighted in the introduction, L-BMAA molecular mechanism of toxicity is largely unknown, with different hypotheses have been formulated. L-BMAA has been reported to induce both acute and chronic neurotoxicity via multiple distinct mechanisms (23, 24, 29, 65). Some experimental results suggest that acute neurotoxicity results from excitotoxic mechanisms dependent on NMDA and glutamate receptors. In contrast, chronic low-dose exposure to BMAA results in alterations in protein folding, aggregation, expression, enzyme activity and neuroinflammation, affecting nervous system function at the cellular level. Although our experimental doses of L-BMAA and exposure time can be considered an acute treatment, we disfavor the excitotoxic hypothesis for two main reasons. The observed toxicity extends beyond neuronal cells, including human fibroblasts (**Figures 1**, **5**) and HEK293 cells (data not shown). Under our experimental conditions, the toxic effect of L-BMAA can be significantly counteracted by the addition of amino acid serine and alanine to the cell growth medium. This last result strongly supports a L-BMAA toxicity mediated by misincorporation during protein synthesis, probably inducing protein misfolding. Consistent with this result, both in cells (66, 67) and in mice (27) protein misfolding appears to be the main L-BMAA toxin effect that, at least in cells, may be counteract by L-serine addiction. Notably, L-serine has been proposed as a potential therapeutic option for ALS (68) and some phase 2 clinical trials (ClinicalTrials.gov Identifier: NCT03580616 for ALS and NCT03062449 for AD) are ongoing. Of course, we are conscious that the molecular mechanism of L-serine neuroprotection can be very complex (69–71) and independent to L-BMAA-mediated neurotoxicity.

Moreover, the potential biomagnification of L-BMAA up the food chain raises pertinent questions regarding dose exposure. For instance, Cox et al. (11) observed a 10,000-fold biomagnification of free L-BMAA and 50-fold biomagnification in total L-BMAA from symbiotic cyanobacteria to cycads to flying fox of the genus *Pteropus mariannus*. These data suggested a mechanism that could produce sufficiently high doses of toxins to induce neurological disease in humans (11, 24, 72, 73).

Among the different molecular pathways altered by cyanobacteria extract or L-BMAA exposure we highlight a significant impairment in autophagy upon cyanobacteria extract or L-BMAA treatment. In fact, two marker of autophagy impairment (LC3B and p62), analyzed by western blot, are upregulated by the treatment (**Figure 2**). In agreement, in NSC-34 cells boosting autophagy rescues the L-BMAA-induced toxicity (74) although a different research suggests that L-BMAA stimulates the chaperone-mediated autophagy activity evaluated by the increase in Lamp2a receptor staining upon L-BMAA treatment (67).

Interestingly, both α-synuclein and TDP-43 are mainly degraded by autophagic mechanisms compared to proteosome pathways (59, 60, 75). Importantly, both proteins accumulate in neuronal cells upon cyanobacteria extract or L-BMAA exposure (**Figure 3**). Moreover, a time course experiment of protein degradation, blocking protein neo-synthesis, strongly evidences that the steady-state synuclein level is altered in the presence of L-BMAA treatment leading to protein accumulation (**Figure 3**) and likely aggregation.

We deeply studied the L-BMAA effect on TDP-43 WT or pathological mutant proteinopathy using different experimental assays. First, we were able to demonstrate that L-BMAA may have a synergistic effect on mutant TDP-43 further increasing the cell toxicity (**Figures 4**, **5**). Moreover, in two different cellular models (SH-SY5Y and human fibroblast) L-BMAA treatment determines a significant TDP-43 mislocalization into the cytoplasm (**Figures 4**, **5**) and a significant accumulation of insoluble aggregates (**Figure 4**), two typical pathological signs of TDP-43 proteinopathy. The effect of L-BMAA on TDP43 is also supported by two different mouse studies. Anzilotti et al. demonstrated that chronic exposure to L-BMAA cyanotoxin induces cytoplasmic TDP-43 accumulation and an ALS phenotype (26) while Arnold et al. showed low dose of L-BMAA associated to a low expression of ALS TDP-43 Q331K mutant results in a motor phenotype that is absent from either lesion alone (27).

Taken together, all our results support a significant role of L-BMAA and LCS-MaCe exposure in α-synuclein and TDP43 proteinopathy and neurodegeneration. Our findings align with the recent discovery that prolonged exposure to L-BMAA in cetaceans, as evidenced by both epidemiological and biochemical observations, triggers distinct indicators of Alzheimer’s Disease (Aβ+ plaques and neurofibrillary tangles in the hippocampus) and TDP-43 proteinopathy (TDP-43 cytoplasmic inclusions in cerebral cortex, midbrain and brainstem) (76). Human beings can be, via dietary sources, chronically exposed to cyanotoxins, that can alter specific cellular pathways leading to protein misfolding and aggregation likely associated to autophagy impairment. Many of these alterations may also determine a chronic mild gut inflammation or exacerbate some innate immune responses that ultimately lead to neurodegeneration. The cyanobacteria-gut-brain axis represents an intriguing and relatively unexplored connection between the microbial world and the intricate communication network within the human body. While the specific mechanisms of interaction remain a subject of ongoing investigation (77), it is hypothesized that cyanobacteria may produce many different compounds or metabolites that can modulate neuronal activity. Understanding the cyanobacteria-gut-brain axis at the neuronal level opens new scenarios for research and unravelling the intricacies of this relationship may provide insights into the development of innovative therapies targeting the gut microbiome to positively influence brain function.

In summary, the cyanobacteria-gut-brain axis represents a fascinating intersection of microbiology and neurobiology, highlighting the interconnectedness of the microbial world with the complex neural networks governing human health. Further exploration of this axis holds the promise of uncovering novel therapeutic strategies and enhancing our understanding of the intricate communication between the gut and the brain.

## Data availability statement

The original contributions presented in the study are included in the article/supplementary material. Further inquiries can be directed to the corresponding author.

## Ethics statement

The human primary fibroblast lines were kindly shared with us by our collaborator Prof. Sandro Orrù, the Department of Medical Genetics of Cagliari University, from a cell bank financed by the foundation AriSLA for “Progetto Eugenio”. The studies were conducted in accordance with the local legislation and institutional requirements. The human samples used in this study were acquired from gifted from another research group. Written informed consent to participate in this study was not required from the participants or the participants’ legal guardians/next of kin in accordance with the national legislation and the institutional requirements. Ethical approval was not required for the studies on animals in accordance with the local legislation and institutional requirements because only commercially available established cell lines were used.

## Author contributions

PS: Writing – original draft, Writing – review & editing, Validation, Methodology, Investigation. GG: Writing – original draft, Writing – review & editing, Investigation. CCi: Writing – review & editing, Writing – original draft, Investigation. MG: Writing – review & editing, Writing – original draft, Project administration, Methodology. BP: Writing – review & editing, Writing – original draft, Conceptualization. AL: Writing – review & editing, Writing – original draft. CI: Writing – review & editing, Writing – original draft, Validation, Funding acquisition, Conceptualization. CCr: Writing – review & editing, Writing – original draft, Funding acquisition, Conceptualization.
